# Changes in Tinnitus Experiences During the COVID-19 Pandemic

**DOI:** 10.3389/fpubh.2020.592878

**Published:** 2020-11-05

**Authors:** Eldré W. Beukes, David M. Baguley, Laure Jacquemin, Matheus P. C. G. Lourenco, Peter M. Allen, Joy Onozuka, David Stockdale, Viktor Kaldo, Gerhard Andersson, Vinaya Manchaiah

**Affiliations:** ^1^Department of Speech and Hearing Sciences, Lamar University, Beaumont, TX, United States; ^2^Vision and Hearing Sciences Research Centre, Anglia Ruskin University, Cambridge, United Kingdom; ^3^Nottingham Biomedical Research Centre, National Institute for Health Research, Nottingham, United Kingdom; ^4^Hearing Sciences, Division of Clinical Neuroscience, School of Medicine, University of Nottingham, Nottingham, United Kingdom; ^5^Nottingham Audiology Services, Nottingham University Hospitals, Nottingham, United Kingdom; ^6^Department of Otorhinolaryngology and Head and Neck Surgery, Antwerp University Hospital, Edegem, Belgium; ^7^Department of Translational Neurosciences, Faculty of Medicine and Health Sciences, University of Antwerp, Wilrijk, Belgium; ^8^Experimental Health Psychology, Maastricht University, Maastricht, Netherlands; ^9^Research Group, Health Psychology, KU Leuven University, Leuven, Belgium; ^10^American Tinnitus Association, Washington, DC, United States; ^11^British Tinnitus Association, Sheffield, United Kingdom; ^12^Department of Psychology, Faculty of Health and Life Sciences, Linnaeus University, Växjö, Sweden; ^13^Centre for Psychiatry Research, Department of Clinical Neuroscience, Karolinska Institutet, Stockholm, Sweden; ^14^Stockholm Health Care Services, Region Stockholm, Stockholm, Sweden; ^15^Department of Behavioral Sciences and Learning, Linköping University, Linköping, Sweden; ^16^Department of Speech and Hearing, School of Allied Health Sciences, Manipal University, Karnataka, India

**Keywords:** COVID-19, public health, tinnitus, coronavirus, understanding, mental health–state of emotional and social well-being, loneliness, social isolation

## Abstract

**Introduction:** The COVID-19 pandemic has disrupted delivery of healthcare, economic activity, and affected social interactions. Identifying and supporting those most affected by the pandemic is required. The purpose of this study was to determine the impact of the pandemic on individuals with tinnitus and to identify mediating factors.

**Methods:** This is a mixed-methods exploratory cross-sectional study, using data collected via an online survey from 3,103 individuals with tinnitus from 48 countries. The greatest representation was from North America (49%) and Europe (47%) and other countries were only marginally represented.

**Results:** Although the study was aimed at those with pre-existing tinnitus, 7 individuals reported having COVID-19 initiated tinnitus. Having COVID-19 symptoms exacerbated tinnitus in 40% of respondents, made no change in 54%, and improved tinnitus in 6%. Other mediating factors such as the social and emotional consequences of the pandemic made pre-existing tinnitus more bothersome for 32% of the respondents, particularly for females and younger adults, better for 1%, and caused no change to tinnitus for 67%. Pre-existing tinnitus was significantly exacerbated for those self-isolating, experiencing loneliness, sleeping poorly, and with reduced levels of exercise. Increased depression, anxiety, irritability, and financial worries further significantly contributed to tinnitus being more bothersome during the pandemic period.

**Conclusions:** These findings have implications for tinnitus management, because they highlight the diverse response both internal and external factors have on tinnitus levels. Clinical services should be mindful that tinnitus may be caused by contracting COVID-19 and pre-existing tinnitus may be exacerbated, although in the majority of respondents there was no change. Additional support should be offered where tinnitus severity has increased due to the health, social, and/or emotional effects of the COVID-19 pandemic. Tinnitus may be more bothersome for those experiencing loneliness, having fewer social interactions, and who are more anxious or worried.

## Introduction

In March 2020, the World Health Organization (WHO) declared the COVID-19 outbreak a global pandemic (1). This pandemic has impacted the lives of millions of people around the globe, causing extraordinary disruption to the delivery of healthcare, economic activity, and social interactions (2). Due to the person-to-person transmission of COVID-19 (3), most countries introduced social distancing restrictions and advised people to stay at home where possible (4).

Although such measures reduced the spread of the virus, they can increase levels of depression and reduce well-being in the general population, as indicated by a systematic review, collating the current evidence (5). This review found lower psychological well-being and higher anxiety and depression compared to before COVID-19. Numerous factors including low-self rated health, poor sleep quality, higher perceived stress load, less family support, and unsteady family income, were associated with this increased risk of depression and anxiety.

Support should be directed toward those at higher risk of reduced well-being during the pandemic, such as those with existing mental health conditions (5). One such at-risk group are those with chronic tinnitus, due to already having an increased risk of reduced emotional well-being, depression, and anxiety (6, 7). Those experiencing tinnitus hear sounds in their head and/or ears in the absence of an external sound (8). It is one of the most frequently occurring chronic conditions, affecting 12–30% of the adult population (9). Although tinnitus occurs in all age groups, older adults have a higher incidence of tinnitus (10). This is also the age group most at risk of severe illness from COVID-19 (11). A complex bidirectional interaction exists between tinnitus and emotional distress, as they can trigger or exacerbate each other (12). Tinnitus frequently spikes or is even initiated during stressful periods (13). Also, due to the pandemic, it is more difficult to receive healthcare for conditions that are not seen as life-threatening, such as tinnitus. The pandemic has been shown to increase fear and worry in the general population (14) and may potentially worsen levels of tinnitus due to the clear relationship between emotional distress and severe tinnitus (15). This may, in turn, increase the societal cost of tinnitus, estimated to be £2.7 billion per annum in the United Kingdom (16). Further research into the impact of COVID-19 on tinnitus is required. Such studies are emerging, for example 122 tinnitus patients from a clinic in Germany indicated that although COVID-19 resulted in increased levels of stress only a small increase in tinnitus distress was found (17). Due to the highly heterogeneous nature of tinnitus (18), it is not known if these experiences would be similar in a non-clinical population. It is also not known if these experiences are unique to those living in Germany. A study targeting the general population experiencing tinnitus from more countries is desirable. The aim of this study was to investigate the impact of COVID-19 and factors that contribute to this impact. The hypothesis for this study is that tinnitus experiences will worsen during the pandemic.

## Methods

### Study Design

A mixed-methods exploratory cross-sectional survey study design was used to explore the impact of the COVID-19 pandemic on experiences of tinnitus. Ethical approval was granted by the Faculty of Science and Engineering Research Ethics Panel at Anglia Ruskin University (Cambridge, UK, reference number FSE/FREP/19/927) for international data collection.

The Equator network Checklist for Reporting Results of Internet e-Surveys was used to report the methods and results of the survey (see Supplementary Material).

### Survey Development

Items for the survey were identified through an iterative process by focusing on the current research identifying factors that could contribute to experiences during the pandemic. A list of possible theme questions was generated by the first author and members of the research team contributed to further themes (VM, DB, DS, JO). The first author drafted the survey consisting of 60 proposed questions. The number of questions were reduced by the research team by considering the appropriateness of each question for a tinnitus population. The final survey comprised of a maximum of 47 closed-ended questions and 3 open-ended questions and took approximately 10–15 min to complete. All questions except the open-ended questions were mandatory, although some of the questions were follow-up questions and only presented if responding “yes” to preceding questions by using skip logic. An example was if answering yes/no to having had COVID-19 symptoms.

The survey captured the following categories:

i) Demographic information such as ethnicity, tinnitus duration, and living situation (16 questions).
ii) Tinnitus severity during the pandemic was measured using the Tinnitus Handicap Inventory-screening version [THI-S; (19)] consisting of 10 questions and based on the full version consisting of 25 questions (20).
iii) COVID-19-related questions regarding following social isolation/distancing guidelines, experiencing COVID-19 symptoms and taking medication (11 questions).
iv) The effects of the restrictions imposed by the COVID-19 situation emotionally and financially (12 questions).
v) Strategies to cope with the current situation such as using social and professional support (10 questions).

The survey went through three stages of review before commencing data collection. Initially, two tinnitus associations (The American and British Tinnitus Associations) and their support groups consisting of individuals with tinnitus, reviewed the questionnaire. This was followed by three independent clinical audiologists reviewing the updated questionnaire. This process attempted to ensure (i) all functionality aspects of the online questionnaire were appropriate, such as progressing to subsequent questions and being able to select multiple responses where appropriate; (ii) the face validity of the questions, to assess whether they clearly capture the aspects they aimed to evaluate; and (iii) the interpretability regarding the wording of the questions (21). The suggestions made also improved the survey flow. In a third stage, the survey was then sent to three individuals experiencing tinnitus, to determine whether it was clear and easy to complete. Subsequently, errors identified were corrected and the comprehensibility of the questions was improved. This process indicated good face-validity of the survey. Although a fully psychometrically validated survey would be preferable, the time sensitive nature of this study did not allow for this and it was not the goal of the study to evaluate the study factor structure or internal consistency thereof.

The final survey items were inputted into Qualtrics (Qualtrics, Provo, UT) and were reviewed by team members to ensure functionality. No randomization of the items was used and respondents were unable to change their responses once submitted. No identifiable data were collected. The questionnaire focused on two main themes: tinnitus experiences, and support required for those with tinnitus during the pandemic. This paper focuses on tinnitus experiences during the pandemic. Results regarding the support required during COVID 19 will be reported separately.

### Survey Translations

To improve accessibility the final English survey was translated into Dutch, Brazilian Portuguese, Portuguese, German, and Swedish. Translation guidelines (22) were followed where possible, but due to the timescale, both forward and backward translation was not possible. The translated versions were cross-checked and corrected by at least two native speakers of each language. Where possible healthcare professionals who had an understanding of hearing-related difficulties, were involved. Linguistic and cultural adjustments to the wording were made to suit each language.

### Survey Distribution

Eligibility criteria included adults aged 18 years or older who provided informed consent. The survey was open to anyone meeting the inclusion criteria. Recruitment was mostly via patient organizations' social media outlets (Twitter, LinkedIn, and Facebook). The American Tinnitus Association (ATA) distributed the survey in the US, the British Tinnitus Association (BTA) in the UK, The Hörselskadades Riksförbund in Sweden, Tuut van Tegenwoordig in Belgium, and Hoorzaken in The Netherlands. The survey was launched on the 29th of April in the UK, the 7th of May in the USA, and the 12th of May 2020 in Belgium, and the Netherlands and later staggered across other European countries and was open for 6 weeks in each location. Online informed consent was required before undertaking the survey and only one submission from each IP address was permitted by the survey software.

### Data Analysis

Data cleaning was initially undertaken to remove cases that did not meet study eligibility due to not having tinnitus, or not completing at least the questions relating to tinnitus on the questionnaire. Data analysis incorporated a mixed approach, including both quantitative and qualitative analysis. The Statistical Package for Social Sciences (SPSS) version 26.0 (IBM Corp, 2019) was used for descriptive statistics, including frequencies, means, and standard deviations. The Chi-Square test was used to test the relationships between categorical variables. Where significant, adjusted residuals were used for *post-hoc* analysis to identify which relationships were significant. Due to multiple testing, the *p*-value was adjusted (Bonferroni) to be significant for *p* = 0.001. Qualitative data from the open questions were analyzed separately using inductive thematic analysis and the themes identified were used to support quantitative analysis.

## Results

### Representation of Individuals With Tinnitus

**Figure 1:**
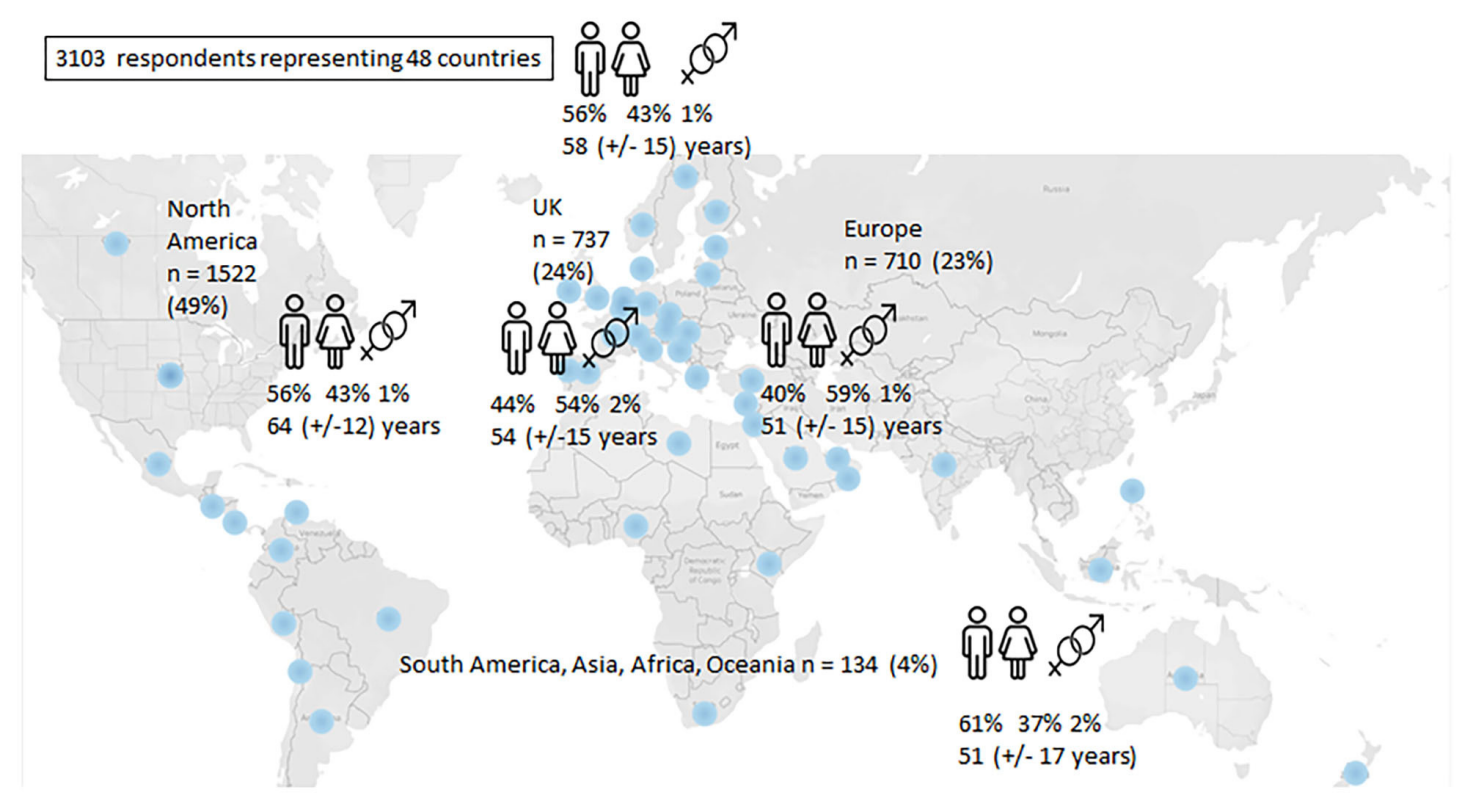
Distribution of respondents.

There were 3,400 respondents. Of these 38 did not provide consent and 259 responses were largely incomplete. The remaining 3,103 respondents represented 48 countries, although some countries were only marginally represented. The highest number (49%) were from North America (USA, Canada), followed by 47% from Europe (European Union and the United Kingdom). For comparative purposes, the whole sample was divided into four groups, a cohort from North America (49%), one from the United Kingdom and Ireland (UK = 24%), a combination of other European countries excluding the UK (e.g., Belgium, The Netherlands, Sweden, Norway, Portugal, France = 23%) and a group representing a combination of other countries located in South America, Oceania, Asia, and Africa (4%) as shown in Figure 1. The age range was 18–100 years with a mean of 58 years (SD:14.0; SE 0.3) with an even gender divide and ethnic distribution where the majority were white (92%) with other ethnic groups (Asian, Black, Hispanic, Indian, or mixed-ethnicity) each representing under 2% of the respondents, respectively.

### Impact of the Pandemic on Tinnitus

**Figure 2:**
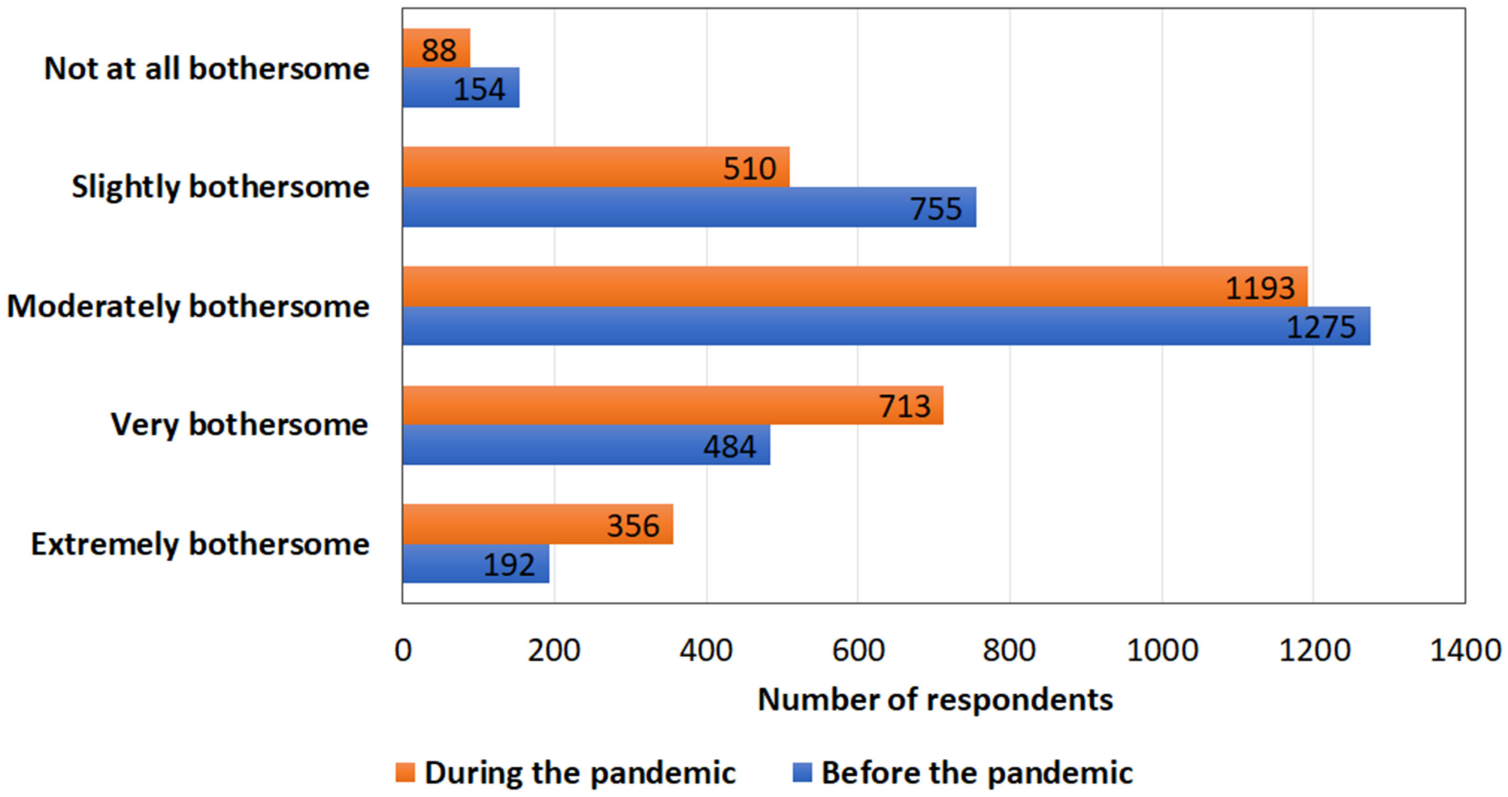
Comparison of how bothersome tinnitus was before and after the COVID-19 pandemic.

The average tinnitus duration was 13.6 (SD:14.0; SE 0.3) years with a range of 0.3–80 years, indicating that most respondents had longstanding chronic tinnitus. When looking at tinnitus severity scores, the mean was 17 (SD:10; SE 0.2, range 0–40) out of 40 (with higher scores indicating more severity) on the Tinnitus Handicap Inventory Screening Version. Tinnitus was rated on average to be more bothersome during the pandemic. It was more frequently rated as “very” (24%) and “extremely bothersome” (13%) compared with before the pandemic (17 and 7%, respectively) as shown in Figure 2. For the majority, these changes were minor in either direction (e.g., from slightly to moderately bothersome). Tinnitus was rated to be stable during the pandemic for 67%, improved for 1%, whereas 32% rated tinnitus as more bothersome. Females (*X*^2^ (15) = 57; *p* = 0.001) and those in age categories below 50 years of age, found tinnitus significantly more bothersome during the pandemic (*X*^2^(15) = 91; *p* = 0.001). Mediating factors related to these reports were explored.

### The Impact of the Health Concerns Stemming From COVID-19 on Tinnitus

**Figure 3:**
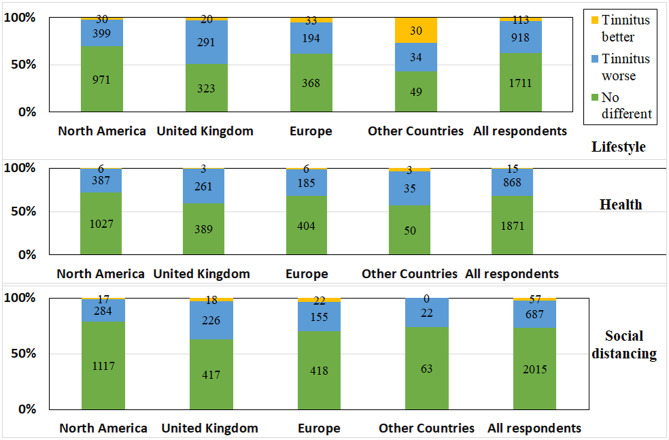
The impact of lifestyle changes, social distancing restrictions, and health concerns during the pandemic on tinnitus experiences.

When asked if health concerns stemming from COVID-19 (e.g., worried about getting ill) affected their tinnitus, 0.5% reported their tinnitus to be improved and 31.5% reported it had worsened (Figure 3).

COVID-19 symptoms were experienced by 8% (*n* = 237/2,952) and 8% (*n* = 249/2,952) were unsure if they had symptoms. Of those experiencing COVID-19 symptoms, tinnitus remained stable for 54% (*n* = 128/237), improved for 6% (14/237) and significantly (*X*^2^ (15) = 345; *p* = 0.001) exacerbated tinnitus for 40% (*n* = 95/237). This was supported by statements such as: “I was too ill to notice at the time, but the tinnitus is definitely much worse now that I'm better enough to notice.” (Female, 55 years, UK). Improvements in tinnitus following having COVID-19 symptoms were explained by “Being focused on getting better pushed the tinnitus issue into the background” (Male, 48 years, USA) and “I noticed the tinnitus less because the virus has shown me there are bigger problems than my tinnitus” (Male, 55 years, Belgium). Only 143/2,952 were tested for COVID-19 and of these 26 (18% of those tested) tested positive and of these, 58% (15/26) reported that their tinnitus was exacerbated by the virus. Being anxious about contracting the virus, also exacerbated tinnitus, as explained: “I'm constantly worrying if I've been exposed. This increased stress makes my tinnitus very loud” (Female, 31 years, UK).

It was not asked specifically whether tinnitus was initiated by having COVID-19, but in free-text, there were mentions of both tinnitus (*n* = 7) and hearing loss (*n* = 4) starting after contracting COVID-19. A respondent explained, “I did not have tinnitus before the virus. It came on when I was ill and is the only thing which has continued afterward” (Female, 52 years, UK) or “having the virus started my tinnitus” (Male, 36 years, The Netherlands).

Only 4 of the 28 (14%) diagnosed with COVID-19 were medicated in hospital, while most took medication at home, such as Ibuprofen, Paracetamol, Lorazepam, Methylprednisolone, Mortin, Tamiflu pills, Tylenol, Robitussin, Azelastine, Salbutamol, or Azythromycine. There were no reports of taking medication such as chloroquine or hydroxychloroquine, which can be ototoxic. Others described taking natural remedies such as Chinese herbs, ginger, garlic, turmeric, honey, lemon, and zinc. Taking medication significantly increased the presence of tinnitus (*X*^2^(8) = 598; *p* = 0.001^*^). When asked which medications affected tinnitus, both prescribed medications e.g., “Steroid medication made my tinnitus worse” (Male, 66 years, USA) and using vitamins to try to boost the immune response against the virus was reported to make tinnitus worse e.g., “I have experienced spikes in my tinnitus when taking vitamins/supplements such as Vitamin D to increase my immune system in response to COVID” (Male, 64 years, USA).

Respondents also had family members who had tested positive for COVID-19 **(**15%; *n* = 37/243), adding further concerns, expressed by comments such as “I think my current tinnitus spike is due to anxiety for myself and my family, since several of us are in the higher risk group” (Male, Norway, 36 years).

Respondents were asked if they had additional health concerns that may put them at risk for developing COVID. Significantly more respondents from North America reported additional health concerns (70%) compared with under 50% from other locations (*X*^2^(6) = 205; *p* = 0.001^*^). The presence of additional health concerns was not related to more bothersome tinnitus (*X*^2^(10) = 222; *p* = 0.02).

### The Impact of Social Distancing Restrictions During the Pandemic on Tinnitus

Social distancing restrictions stemming from COVID-19 did not affect tinnitus for 73% of respondents, improved it for 2%, and exacerbated tinnitus for 25% (Figure 3). Those reporting a positive impact, explained the reasons as follows: “I'm less frustrated not being in large crowds” (Female, 38 years, UK). In addition to the tinnitus being exacerbated, social distancing also made listening hard. This was explained by statements such as: “Having to understand 6 feet away and through a mask is so much harder and raises my irritation levels” (Male, 52 years, USA).

**Figure 4:**
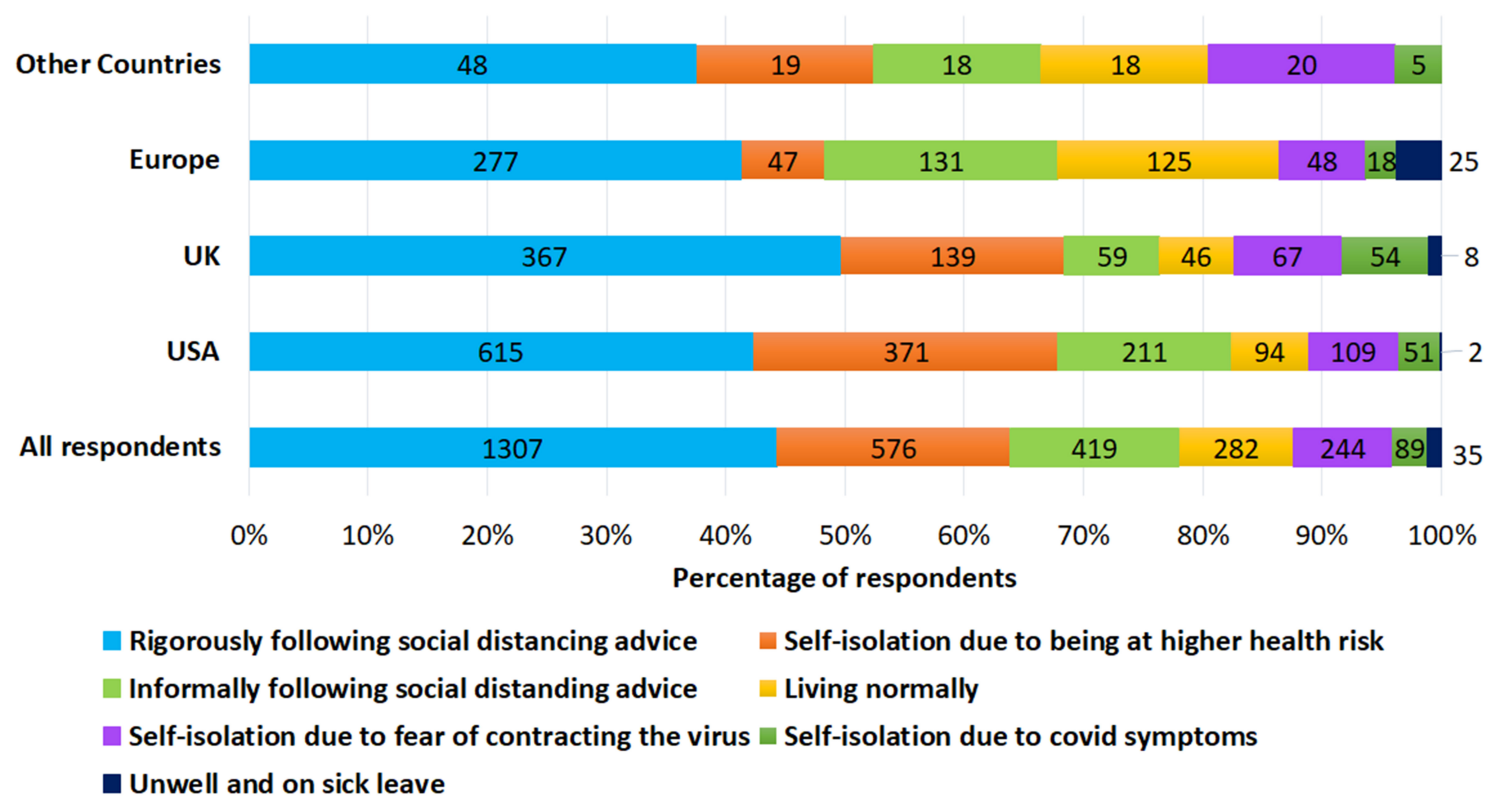
The following of social distancing advice across different locations.

The impact of social distancing restrictions varied significantly between countries (*X*^2^ (12) = 214; *p* = 0.001^*^) and had a significantly greater impact (*p* = 0.001^*^) in the UK (34%) compared with North America (20%). Social distancing advice was followed by 44% of respondents, particularly from the UK (50%), as shown in Figure 4. Tinnitus was significantly more bothersome for those who were self-isolating (*X*^2^ (35) = 550; *p* = 0.001).

#### The Impact of Social Interactions

**Figure 5:**
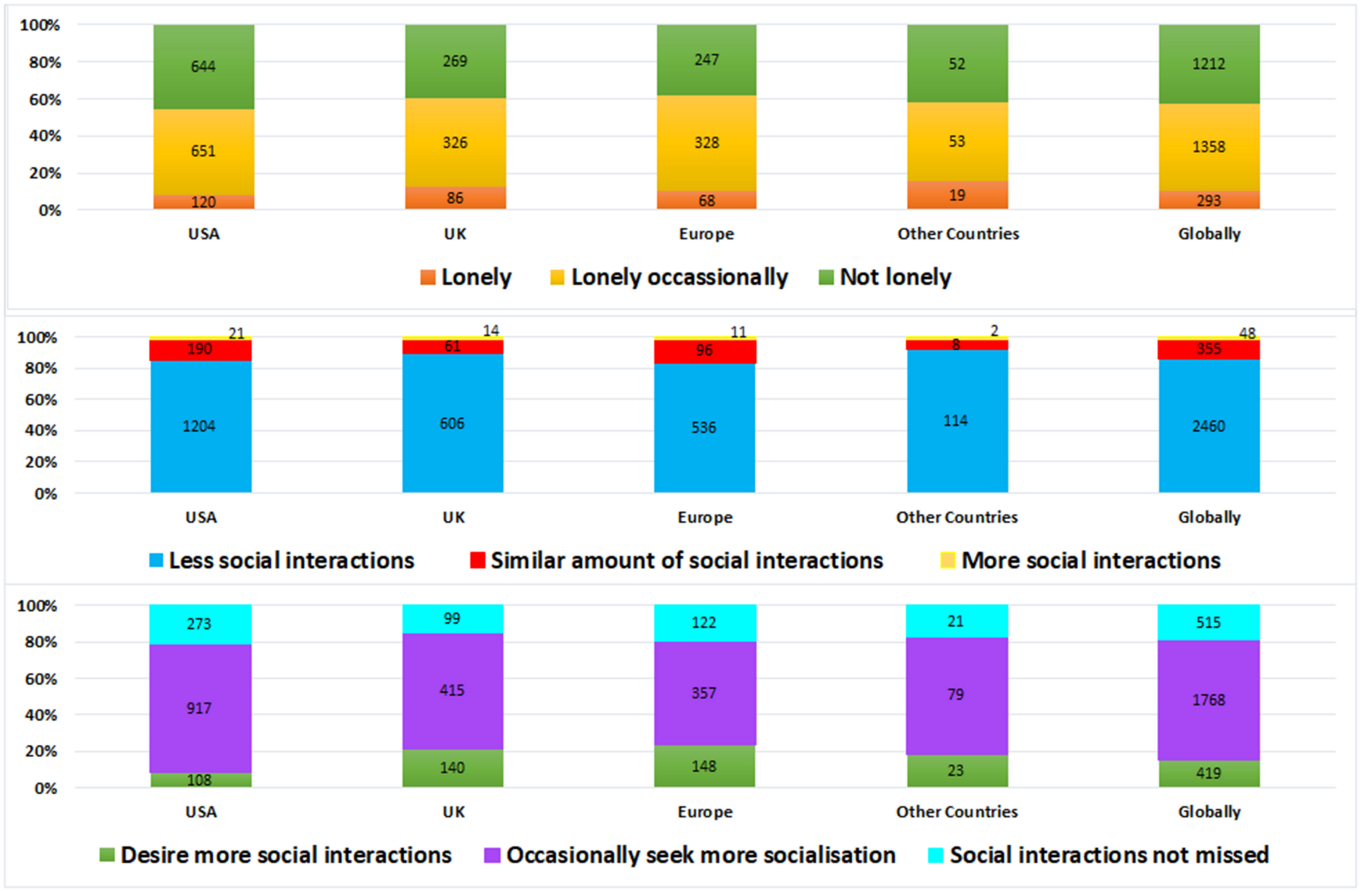
The frequency of social interactions, desire for more interactions and loneliness experienced during the pandemic across different locations.

Figure 5 indicates that 86% of respondents reported fewer social interactions, 12% had a similar amount and 2% had more social interactions. More social interactions were desired by 84% of respondents, particularly in the UK as explained: “I notice my tinnitus more because I am stuck in my house all alone with nobody to speak to” (Female, 45 years, UK).

#### The Impact of Loneliness

When asked if respondents feel lonely because of pandemic related lockdown, most respondents (58%) reported being lonely. Tinnitus was significantly more bothersome for those reporting loneliness (*X*^2^ (15) = 1,213; *p* = 0.001). Experiences of loneliness may have been amplified during the pandemic due to the lockdown measures in place, as explained by the statement “So much time alone has just made me more aware of the tinnitus” (Female, 71 years, USA). When comparing locations, significant differences were found (*X*^2^ (9 = 35; *p* = 0.003^*^) as those in North America reported significantly (*p* = 0.001^*^) less loneliness (54%) than those in Europe (62%), the UK (60%), and combined other countries (58%).

### The Impact of Lifestyle Changes Due to the Pandemic on Tinnitus

Lifestyle changes stemming from COVID-19 did not affect tinnitus for 62% of respondents, exacerbated tinnitus for 34%, and improved it for 4%. There were significant differences between locations (*X*^2^ (12) = 232; *p* = 0.001^*^), as lifestyle changes impacted those in the UK (46%) significantly more (*p* = 0.001^*^) than those in North America (29%), as seen in Figure 3. From the free text responses, some respondents reported tinnitus starting during the pandemic and assumed this was related to lifestyle changes saying “No one knows why my tinnitus started, it may be from staying at home, being out of routine or the stress” (Female, 44 years, UK).

#### The Impact of Living Demographics

To identify how lifestyle changes may have been affected, respondents were asked about their living demographics. The majority of respondents live in a city (48%, *n* = 1,483), a town (29%, *n* = 896) or small town (12%, *n* = 370) whereas 11% (*n* = 354) live rurally or in the countryside. Towns and cities were quieter than they would have been used to, which may have altered tinnitus experiences. Tinnitus being more noticeable due to life being quieter was often mentioned e.g., “I am now more aware of the tinnitus as my household is very quiet” (Female, 59 years, UK). For some being at home resulted in exposure to more noise, making tinnitus worse, such as “Increased noise from power tools/lawn equipment and kids playing on motorized toys have made my tinnitus worse” (Female, 43 years, USA).

The majority of respondents had access to a garden or park during the pandemic (89%, *n* = 2,762). The cohort from South America, Asia, Africa, and Oceania reported significantly less (*p* = 0.001^*^) outdoor spaces (65%) in comparison to 88% from North America, 90% from Europe, and 94% from the UK. Having access to nature was reported to have a positive impact on tinnitus, explained by “I've been furloughed, so I am enjoying relaxing and being in nature more” (Male, 69 years, UK), although this association was not significant.

#### The Impact of the Pandemic on Sleep

Sleep problems such as waking up earlier or having less restful sleep were reported by 67% respondents, with 46% (*n* = 1,819) describing lower sleep quality, explained as “I've not been able to sleep because of a change in routine and worrying, which makes my tinnitus louder” (Male, 56 years, UK). More troubled sleep was related to tinnitus being significantly more bothersome (*X*^2^(5) = 113; *p* = 0.001). Better sleep was reported by 6% (*n* = 221), for example, “Being at home means I have more time to sleep, meditate, do yoga, and eat healthy meals which all help me” (Female, 42 years, Canada).

#### The Impact of Exercise and Diet

Compared with before the pandemic, 38% of respondents reported doing more exercise and 46% reported doing less exercise, which contributed to tinnitus being significantly more bothersome (*X*^2^(15) = 323; *p* = 0.001). When comparing current diet with that before the pandemic, it was similar for 59%, healthier for 17%, and less healthy for 24%, which also added to tinnitus being significantly more bothersome (*X*^2^(15) = 326; *p* = 0.001). An increased intake of caffeine and alcohol was reported to alter tinnitus as follows, “I find myself drinking more coffee which makes the ringing stronger” (Female, 74 years, USA) or “I'm drinking more alcohol and this makes my night-time tinnitus worse” (Female, 41 years, UK).

### The Impact of the Pandemic on Emotional State

**Figure 6:**
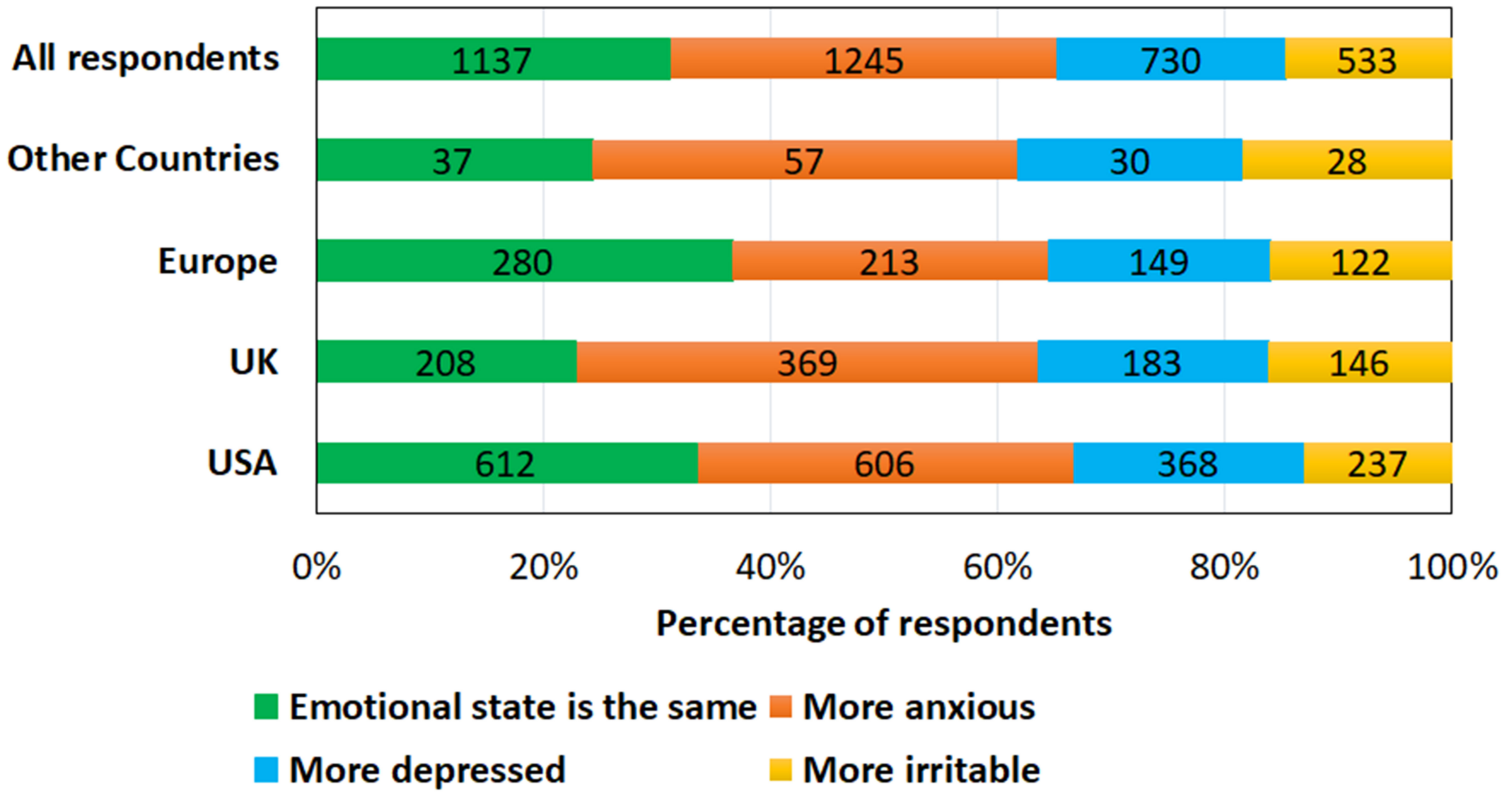
Comparison of emotional state during the COVID-19 pandemic across different locations.

Of the respondents, 34% reported being more anxious, 20% more depressed, 15% more irritable whereas 31% reported no change in their emotional state (Figure 6). Tinnitus was significantly more bothersome for those feeling more sad or depressed (*X*^2^(5) = 58; *p* = 0.001); more anxious (*X*^2^(5) = 107; *p* = 0.001); and more irritable (*X*^2^(5) = 48; *p* = 0.001). Increased anxiety negatively impacted tinnitus as explained: “There is a lot of added stress and anxiety that make me less able to tolerate the tinnitus” (Male, 69 years, USA) or “So many more anxieties with household appliances breaking that can't be fixed, worrying about food supply, worrying about the virus. Any kind of worries has a negative effect on tinnitus” (Male, 73, UK). Frustration was often mentioned as impacting negatively on tinnitus such as “I'm frustrated with the confinement which I think makes my tinnitus seem extra loud” (Female, 72 years, USA), as well as relationship worries, “My tinnitus is really bad now. Maybe from relationship worries caused by my husband working from home. My cortisol level is on permanent red alert, it feels like in a war zone” (Female, 52 years, UK).

Emotional well-being experiences varied across locations as seen in Figure 6 with those from the UK reporting more anxiety/depression and irritability during the pandemic (77%) and Europeans the least (67%).

#### The Impact of Financial Worries

The majority of respondents (51%) reported no financial worries, 41% were somewhat worried, and 8% were very worried about the impact of COVID-19 on finances. Tinnitus was significantly more bothersome (*X*^2^(15) = 345, *p* = 0.001) where financial worries were reported. This was supported by statements such as “As a self-employed wedding photographer, all work has been canceled for the foreseeable future, and I have a massive loss in income which I think is contributing to my tinnitus being worse” (Female, 58 years, USA). Loss in investment income was a further factor causing anxiety “I'm retired and my investment income has dropped dramatically, causing stress” (Female, 63, USA).

**Figure 7:**
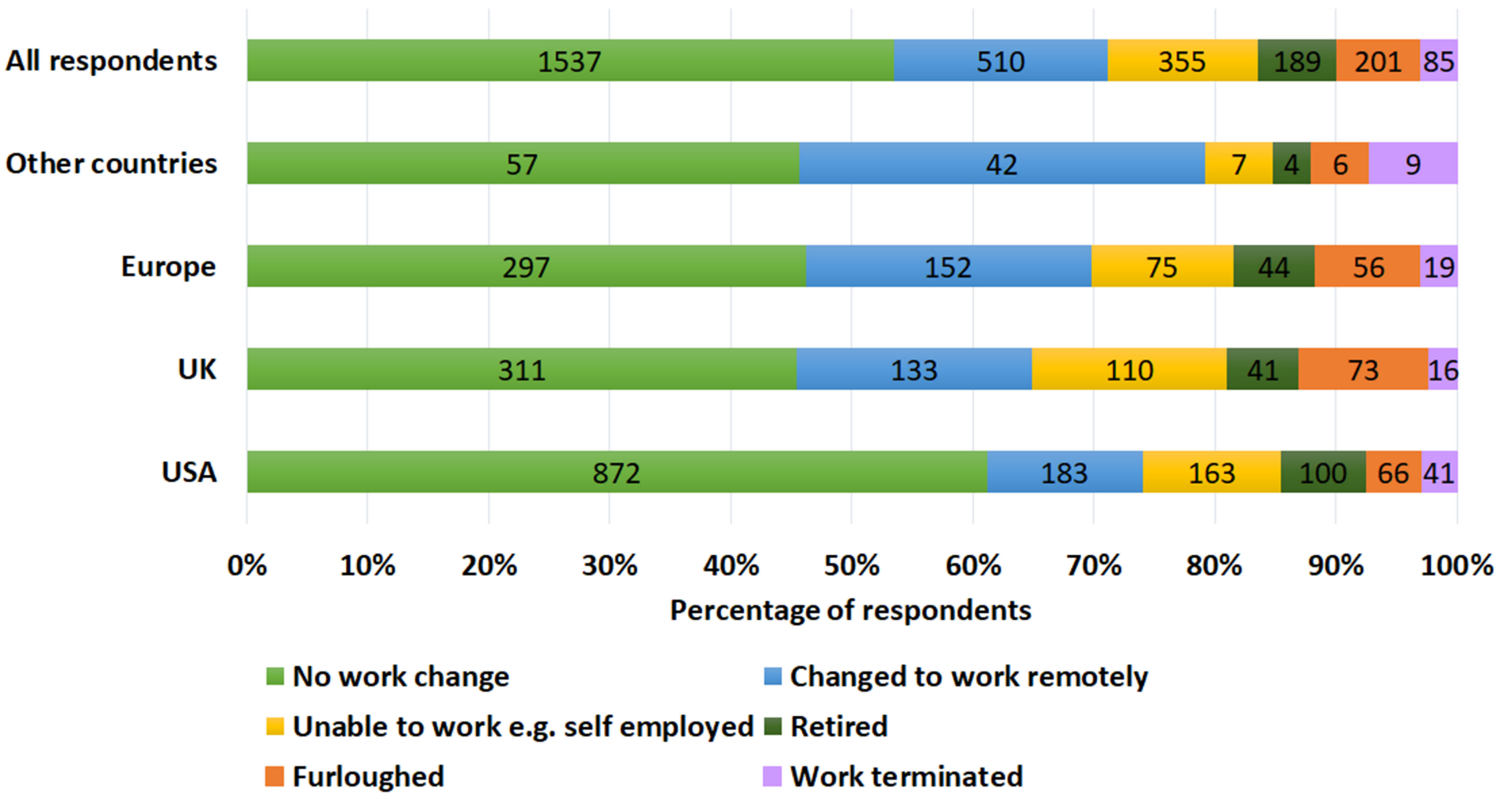
Comparison of changes to work patterns during the COVID-19 pandemic across different locations.

Looking into reasons for financial worries, respondents were asked about changes in their employment situation as seen in Figure 7. Changes in employment patterns altered sound exposure which had a positive effect on tinnitus for some, supported by statements such as “working at home in quiet, instead of a crowded environment gives me less tinnitus” (Male, 53 years, France) and a negative effect for others, as explained “I think the increase in tinnitus is due to less noise from traffic and a busy office” (Female, 54 years, UK).

Although some reported benefits to working from home, there were numerous comments regarding how this change exacerbated tinnitus, such as “I now have a much higher workload, I'm more tired, this increases my tinnitus” (Female, 65 years, USA). Working from home was associated with increased stress which aggravated tinnitus, for example, “Struggling to work at home with nobody to ask when I'm stuck makes me panic and then the tinnitus a lot worse” (Female, 46 years, UK). Working from home together with home-schooling and an increase in household chores, were further factors affecting tinnitus, explained by “It is very busy because my child is at home, and in addition to work, housekeeping, teacher and entertainment. There is little time to relax” (Female, 36 years, The Netherlands).

Participants were asked to explain their responses regarding their current tinnitus experiences in free text. Thematic analysis for these responses identified diverse and overlapping factors that contributed to these experiences as are summarized in Table 1. Overall tinnitus was more bothersome during the pandemic than before the pandemic.

**Table 1:** Factors related to tinnitus being stable, better, or exacerbated during the pandemic that were identified through thematic qualitative analysis of free text responses.

	**Health-related factors**	**Social distancing restrictions**	**Lifestyle changes**	**Emotional state**
Tinnitus exacerbated (31%)	• Health concerns • Family health concerns • Concerned about contracting the virus • Effects of having the virus • Future healthcare • Difficulty accessing healthcare • Reduced ability to access hearing healthcare • Taking medication/ vitamins • Fluctuations in the tinnitus sounds heard	• Rigorously following social distancing advice • Fewer engagements • Fewer social interactions • Housebound • Loneliness • Listening difficulties	• Less exercise • Noisier at home • Too quiet • Increased alcohol intake • Increased caffeine intake • Diet less healthy than prior to the pandemic • Higher workload • Busier • Decreased activity levels • Less exercise compared with before the pandemic • Poor sleep	• Frustrations • Relationship problems • Stress, worrying and anxiety • More depressed • More irritable • Financial worries • More jobs (work, schooling, household) • Lack of relaxation time • Work terminated or furloughed
Tinnitus better (2%)	• Reframing problems • Fighting the virus	• Reduced listening frustration	• Healthier than prior to the pandemic • Increased relaxation • Sleeping better • More peaceful lifestyle • Quieter • More time in nature • More exercise • Better diet	• Working from home
Tinnitus stable (67%)	• No additional health concerns • Tinnitus not severe • Not had virus • Family healthy	• Acceptance of new routine • Not self-isolating • Continuing social interactions	• Access to outdoor spaces • Diet unchanged	• No additional mental health concerns • No financial changes • Similar work patterns

## Discussion

This survey, completed by 3,103 individuals from 48 countries, provides insights regarding tinnitus experiences during the COVID-19 pandemic. Experiencing COVID-19 symptoms did not impact upon tinnitus for 54%, improved tinnitus experiences for 6% and exacerbated it for 40% of the respondents. Of the 26 that were tested positive for COVID-19, 58% reported that their tinnitus was exacerbated by the virus. For some, focusing on surviving the virus helped reframe problems and push tinnitus into the background of their thoughts. Having COVID-19 was antidotally reported to initiate tinnitus (*n* = 7) and hearing loss (*n* = 4). Tracking and managing hearing-related changes due to having the COVID-19 will be important as clinical services resume (23). A rapid systematic review indicated only a few reports of audio-vestibular symptoms in confirmed COVID-19 cases but emphasized the need for understanding the longer-term risks (24). A change in hearing status has been reported by 1 in 10 COVID-19 adults after discharge from one hospital in Manchester in the UK (25) highlighting the need for further monitoring. This is particularly important for those who may have been treated with ototoxic medications (26).

Overall tinnitus was rated as more bothersome during the pandemic than before. An increase in the number of individuals complaining of heightened tinnitus severity since access to their clinics was reinstated post lockdown has also been reported in Italy (27). Those who were female and younger were more likely to find tinnitus more bothersome. From the explanations provided, it appears this may be partly attributed to greater lifestyle changes in these groups during the pandemic, such as changes in employment, increased childcare, and household responsibilities. A study involving tinnitus patients at a clinic in Germany found that COVID-19 brought increased grief, frustration, stress, and nervousness, although there was only a small increase in tinnitus distress compared with 2 years prior to lockdown. Tinnitus distress was worst for those with high neuroticism, indicating that both external and internal factors contribute to tinnitus distress (17).

Lifestyle changes imposed by the pandemic appeared to be one of the factors making tinnitus worse, as reported by a third of the respondents and conversely the greatest factor in improving tinnitus for a small minority. The same experience such as working from home for some worsened tinnitus, and for others, improved tinnitus, highlighting the heterogeneous nature of tinnitus experiences. Being away from all the noise associated with crowded places lowered tinnitus levels for some respondents, whereas due to it being quieter working from home, many found their tinnitus was more bothersome. Tinnitus is often reported to be more noticeable in quiet and has led to tinnitus treatments often using some form of background noise to help prevent the starkness of the tinnitus percept (28). Others reported that working from home exposed them to more noise than usual from neighbors, electrical tools, and children, which aggravated tinnitus. Those with tinnitus often report trying to avoid noisy situations (29).

Diverting attention by focusing on other activities and being physically active is a common strategy used to cope with tinnitus (29). Thus, not being distracted by these activities due to the lockdown restrictions was found to exacerbate tinnitus. Being too busy, however, resulted in more stress and less sleep, which appeared to aggravate tinnitus. The majority of respondents reported lower sleep quality, more trouble sleeping, waking up more during the night, and being less rested, which was significantly associated with tinnitus being worse, whereas 6% reported better sleep due to having more time to relax. Tinnitus related distress has also previously been related to insomnia (30). Some respondents reported having a more relaxed and peaceful lifestyle that made their tinnitus less noticeable. This highlights the importance of relaxation techniques and mindfulness training during the provision of tinnitus therapies (31). Similar to the present survey, it has previously been reported that tinnitus is worse in both quiet and noisy situations when stressed, and due to lack of sleep (32). Tinnitus was significantly worse for those with less healthy diets. Drinking more coffee, alcohol, and taking more supplements to build immunity were reported to negatively impact tinnitus for some respondents. Although no clear relationship exists, previous studies have also noted that caffeine may exacerbate tinnitus (32). Diversity in the factors found to improve or worsen tinnitus across respondents, amplify the difficulty in attempting to subtype tinnitus (33).

Increased levels of anxiety, depression, and irritability were reported. These emotional factors are associated with exacerbated tinnitus annoyance (15). Different worries and frustrations exacerbated these negative feelings including more relationship problems due to being confined, worrying about food supply, and concerns regarding contracting the virus. Financial worries also made tinnitus significantly worse, due to being made redundant, being furloughed, and reduced value of investments. These findings mirror a recent systematic review regarding the impact of COVD-19 on mental health that indicated lower psychological well-being and higher levels of anxiety and depression in the general public compared to before COVID-19 (5). This emphasizes the support needed for both those experiencing tinnitus and the general population to reduce anxiety and depression and improve well-being during the pandemic. This support is likely to be needed after the pandemic as well, due to the likely continued financial difficulties and impact on many aspects of society. A common theme was that concerns regarding contracting the virus made tinnitus worse. These concerns could trigger additional fear-avoidance behaviors (34), where respondents rigorously followed social distancing advice or self-isolated, to avoid risks of contracting COVID-19, despite a desire for more social interactions. This may have, however, contributed to fewer social interactions and feeling lonelier, which was associated with tinnitus being significantly worse. This aligns with previous literature indicating that social distancing (35) and self-isolation results in negative psychological effects (36) and poorer mental health outcomes (37). When social interactions were possible, having to stand further away and follow the conversation while people were wearing a mask was reported to make conversing more difficult.

Around half of the respondents reported occupational changes due to the pandemic. Having to wear headphones and participate in video conferencing calls was often mentioned as negatively impacting tinnitus. Neck strain from working on computers all day had an additional negative impact. For others, juggling homeschooling, work, and more household tasks resulted in more stress and little relaxation time. For some, working remotely meant not commuting and having more time to relax, which positively impacted tinnitus.

This survey has highlighted how emotional state, health concerns, social contact, and lifestyle contribute to tinnitus distress. The relationship between tinnitus and these internal and external factors is complex. Interestingly, respondents living in the UK were most likely to report how the pandemic negatively affected their tinnitus, which may be linked to the rapid spread of the virus and the high death rate in the UK during the time of the survey. The knowledge of these factors is of value during the clinical management of those with tinnitus and special attention should be placed to fully explore health, social, occupational, and emotional factors that may contribute to tinnitus severity.

### Limitations and Future Studies

Although this study attempted to capture a range of views regarding the impact of the COVID-19 pandemic on tinnitus experiences world wide, it is not fully representative. Most responses were from the USA and Europe. Furthermore, not all ethnic minority groups are represented as white ethnic representation dominate this survey, although similar ethnic dominance has previously been found from other surveys [e.g., (38)]. A further drawback is that it was not possible to use a psychometrically validated questionnaire for this survey due to the time sensitive nature of the study. The timescale did also not allow scope to perform both forward and backward translation and fully pilot the translated questionnaires. The study design did not allow for longitudinal comparison as there were no comparative scores (e.g., of tinnitus severity) before the pandemic. A further limitation of this study is that there was no clinical evaluation of the respondents. Findings are based only on self-reported survey data without any clinical data. Additionally, standardized self-reported questionnaires were not included to measure levels of anxiety, depression, hearing loss, and hyperacusis. Moreover, limited data were collected regarding dimensions of the tinnitus, such as its location, type, and number of sounds heard. The cross-sectional design limits causal relationships, although this was countered by the use of direct quotes to formulated probable paths for causal relationships. This study also did not capture the population who may have developed tinnitus due to COVID-19 or during the pandemic, as those with chronic tinnitus were targeted. Furthermore, it is not presently known if the tinnitus would become chronic if it developed after having COVID-19, and therefore should be monitored. However, as there were a few accounts of tinnitus and hearing loss initiation after contracting COVID-19, exploring how frequently this happened in those with COVID-19 symptoms should be investigated. Identifying how people with tinnitus are coping during the COVID-19 pandemic and what support is required is important and is currently being investigated. Future studies should focus on whether these effects change over time as the true impact of COVID-19 experienced or as lockdown restrictions are lifted.

### Clinical Implications

As the COVID-19 pandemic may remain for the foreseeable future, the health, social, and emotional implications are likely to continue for some time. Ways of supporting those experiencing the most profound effects, such as individuals who are socially isolated, should be prioritized by patient organizations and support services. Although in-person support may be restricted for the foreseeable future for non-essential healthcare concerns, online interventions such as Internet-based cognitive behavioral therapy (39) or other remote tinnitus interventions could be valuable for those in need. Investment should be increased for services that provide tinnitus support. A careful case history is required as survivors of COVID-19 treated with certain ototoxic medications, such as chloroquine or hydroxychloroquine, may be at an increased risk for developing hearing loss, tinnitus, or balance problems (40). Those who have had COVID-19 should be monitored for changes in hearing-related problems, such as initiation or worsening of tinnitus. There is most likely also a cohort of patients who experienced an onset of tinnitus during this period and who will need access to clinical care for their tinnitus. Those who are most socially isolated, lonely, as well as those with poor sleep, are at most risk for tinnitus severity increasing. It highlights the heterogeneity of tinnitus with many people responding well, and others struggling during the pandemic and in need of additional support.

## Data Availability Statement

The raw data supporting the conclusions of this article will be made available by the authors, without undue reservation.

## Ethics Statement

The studies involving human participants were reviewed and approved by Anglia Ruskin University, Cambridge, UK, reference number FSE/FREP/19/927. The patients/participants provided online informed consent to participate in this study.

## Author Contributions

This study was conceptualized by EB and designed by EB, VM, DB, PA, DS, and JO. Data collection was by EB, VM, GA, VK, LJ, ML, JO, and DS. Data analysis and interpretation and drafting the article was done by EB. All authors critically revised the article and approved the version to be published.

## Conflict of Interest

The authors declare that the research was conducted in the absence of any commercial or financial relationships that could be construed as a potential conflict of interest.
