# Supplementary material for: Changes in Tinnitus Experiences During the COVID-19 Pandemic
Source: Front Public Health. 2020 Nov 5;8:592878. doi: 10.3389/fpubh.2020.592878 (PMC7676491; doi:10.3389/fpubh.2020.592878)
Supplement: Supplementary file 1 [file Table_1.DOCX]

Survey questions

How old are you in years? (e.g. 55)

________________________________________________________________

What gender are you?

- Male
- Female
- Gender diverse
- Prefer not to say

Where are you presently living?

- United Kingdom
- Ireland
- Germany
- The Netherlands
- France
- Italy
- Sweden
- Austria
- Portugal
- Belgium
- Greece
- Switzerland
- Asia
- Australia
- New Zealand
- South America
- South Africa

Another country. Please specify:

What is your ethnicity?

- Prefer not to say
- White
- Black
- White and Black
- White and Asian
- Any other Mixed/ Multiple ethnic background
- Indian
- Pakistani
- Bangladeshi
- Chinese
- Any other Asian background
- Arab
- Any other ethnic group. Please state a

Where are you currently staying?

- A city
- A town
- A village
- The countryside
- Other. Please state ________________________________________________

At present, do you have access to a garden, nature or a green space?

- No
- Yes

Have you been diagnosed by a health professional with: (Select all that apply):

- NONE OF THESE
- Hypertension
- Cardiac disease
- Chronic allergy
- Chronic neck and/or back pain
- Osteoarthritis
- Chronic bronchitis
- Diabetes
- Stroke
- Chronic migraine and/or other frequent chronic headaches
- Cancer
- Multiple sclerosis
- Thyroid disease
- Head Injury
- Temporomandibular joint disorder

Have you ever been diagnosed with a mental health condition by a health professional with: (select all that apply)

- No
- Depression
- Anxiety
- Other psychiatric disorders

How are you managing this mental health condition? (Select all that apply):

- Not doing anything
- With medication
- With an online intervention or tool
- Seeing a professional
- Other, please state ________________________________________________

Do you have: (select all that apply):

- NONE OF THESE
- Tinnitus (the term for hearing a sound in the head and/or ears in the absence of any external sound, lasting for more than five minutes at a time)
- Hearing difficulties/ problems
- Hearing difficulties/ problems only in background noise
- Hyperacusis (the term for reduced tolerance to sound)
- Misophonia (the term for severe sensitivity to specific soft sounds)

How long have you had tinnitus? State the number of years (e.g. 0.5 or 7):

________________________________________________________________

Answer the following questions regarding how bothersome your tinnitus is on a scale of 0-5:

|  | Not at all bothersome | Slightly bothersome | Moderately bothersome | Very bothersome | Extremely bothersome |
| --- | --- | --- | --- | --- | --- |
| Prior to the coronavirus? |  |  |  |  |  |
| At present, following the coronavirus outbreak |  |  |  |  |  |

During the last week:

|  | Yes | Sometimes | No |
| --- | --- | --- | --- |
| Because of your tinnitus is it difficult for you to concentrate? |  |  |  |
| Do you complain a great deal regarding your tinnitus? |  |  |  |
| Do you feel as though you cannot escape your tinnitus? |  |  |  |
| Does your tinnitus make you feel confused? |  |  |  |
| Because of your tinnitus, do you feel frustrated? |  |  |  |
| Do you feel that you can no longer cope with your tinnitus? |  |  |  |
| Does your tinnitus make it difficult for you to enjoy life? |  |  |  |
| Does your tinnitus make you upset? |  |  |  |
| Because of your tinnitus do you have trouble falling asleep at night? |  |  |  |
| Because of your tinnitus, do you feel depressed? |  |  |  |

Which device/s do you use to help you with your hearing-related difficulties and/or tinnitus? Select all that apply:

- None
- Hearing aid (one ear)
- Hearing aid (both ears)
- Wearable sound generator for tinnitus (one ear)
- Wearable sound generator for tinnitus (both ears)
- Cochlear implant (one ear)
- Cochlear implant (both ears)
- Bone anchored hearing aid (one ear)
- Bone anchored hearing aid (both ears)
- Hearable or smart earbud (one ear)
- Hearable or smart earbud (both ears)

Have you sought help or treatment for your tinnitus/ hyperacusis/ misophonia in the past or at present? (Select all that apply):

- No
- Yes, sought advice/ help from a professional, such as an audiologist or ENT Consultant
- Yes, had group therapy
- Yes, undertook an internet intervention
- Yes, self-help support such as reading books
- Yes, help via a charity or non-profit organization/ helpline

Which professional have you consulted regarding your tinnitus/ hyperacusis/ misphonia? (Select all that apply:)

- General practitioner/ primary care doctor
- Ear Nose and Throat Consultant (ENT)/ Audiovestibular physician using national healthcare (NHS)
- Ear Nose and Throat Consultant (ENT)/ Audiovestibularphysician privately
- Audiologist/ hearing therapist using national healthcare (NHS)
- Audiologist/ hearing therapist privately
- Psychologist/ Psychiatrist
- Other. Please explain

Prior to the coronavirus pandemic, were you involved in a tinnitus support group?

- No
- Yes, in-person meetings
- Yes, online support or information from the support group
- Involved in tinnitus online discussion forums

Were you negatively affected by the inability to attend support group meetings in person due to social distancing restrictions?

- No
- Yes
- Partly, as I now attend online support group meetings

Are you currently:

- Self-isolation (if you or someone in your household has symptoms)
- Self-isolation due to being at higher risk of contracting the virus (due to other illnesses or older age)
- Self-isolation due to fear of contracting the virus (no social interactions outside your household)
- Rigorously following social distancing advice (reducing social interactions with others)
- Informally following social distancing advice
- Unwell and on sick leave
- Living normally (e.g. continuing to go to a place of work and do the same activities as prior to the pandemic)

Have you had coronavirus symptoms?

- No
- Unsure
- Yes

Have you been tested for the coronavirus?

- No
- Yes, and diagnosed with the coronavirus
- Yes and not diagnosed with the coronavirus

Do you suspect than anyone in your household has been infected by the coronavirus?

- No
- Yes, and diagnosed with the coronavirus
- Yes and not diagnosed with the coronavirus

Have you taken any medication due to the coronavirus?

- No
- Yes. Taken medication at home. Please provide the name of the medication: ________________________________________________
- Yes. Been hospitalized/ on a drip. Please provide the name of the medication: ________________________________________________

Did the medication effect your **hearing**?

- No
- Yes, made it worse in one ear
- Yes, made it worse in both ears
- Yes, but only for a short period of time
- Other, please explain ________________________________________________

Did the medication effect your tinnitus or cause **tinnitus**?

- No
- Yes, initiated the onset of tinnitus
- Yes, made the tinnitus worse
- Yes, made it worse for a short period of time
- Other, please explain ________________________________________________

Do you suspect having coronavirus or these symptoms affected your **tinnitus**?

- No
- Yes it is worse
- Yes, it is better
- Other. Please explain: ________________________________________________

Have health-related worries related to the coronavirus situation, affected your **tinnitus** in some way?

- No
- Yes, it is worse
- Yes, it is better
- Other. Please explain: ________________________________________________

Have lifestyle changes associated with containing the coronavirus affected your **tinnitus**?

- No
- Yes, it is worse
- Yes, it is better
- Other. Please explain: ________________________________________________

Have the requirements of social distancing affected your **tinnitus** in any way?

- No
- Yes, it is worse
- Yes, it is better
- Other. Please explain: ________________________________________________

Have health-related worries related to the coronavirus situation affected your **sound tolerance** issues in some way?

- No
- Yes, it is worse
- Yes, it is better
- Other. Please explain ________________________________________________

Have lifestyle changes associated with containing the coronavirus affected your **sound tolerance** issues in some way?

- No
- Yes, it is worse
- Yes, it is better
- Other. Please explain ________________________________________________

Have the requirements of social distancing affected **sound tolerance** issues in some way?

- No
- Yes, it is worse
- Yes, it is better
- Other. Please explain ________________________________________________

Has the coronavirus pandemic situation resulted in any **positive experiences** related to your tinnitus or sound-tolerance issues?

- No
- Yes. Please explain ________________________________________________

Has your employment changed due to the coronavirus?

- No
- Yes, furloughed
- Yes, laid off/ terminated
- Yes, now working remotely
- Other, please state ________________________________________________

How worried are you about your financial security due to the coronavirus?

- Not at all worried
- Somewhat worried
- Very worried

Has the coronavirus pandemic affected your emotional state compared with before the corona pandemic? Select all that apply

- No, my emotional state is the same as before
- Yes, I feel more depressed/sad
- Yes, I am more anxious/tense
- Yes, I am more irritable/angry

Do you feel lonely in your current situation?

- Hardly ever
- Occasionally
- Most of the time

Do you desire more social interaction at present?

- No
- Occasionally
- Most of the time

How much social contact do you have now compared with before the pandemic?

- Less contact
- Similar contact
- More contact

 How does your current exercise routine compare with your routine prior to the pandemic?

- Less exercise
- Similar amount of exercise
- More exercise

What resources are you drawing on to help cope with your present situation, including strategies to ease stress? (tick all that apply)

Due to current circumstances, has your diet become:

- Healthier
- The same as before
- Been less healthy

Due to current circumstances, has your sleep been affected? Select all that apply:

- No
- I am sleeping better
- I am having more trouble sleeping
- I am less rested (lower sleep quality)
- I wake up earlier than usual
- I wake up more during the night

Contacting friends/ family

- Relaxation
- Mindfulness
- Yoga/ Pilates
- Exercising indoors
- Exercising outdoors
- Spending time outdoors
- Other. Please explain

Due to current circumstances, would you say you:

- Never drink alcohol
- Consume less alcohol than normal
- Drink similar amounts of alcohol
- Drink more alcohol than normal
- Engage in more substance abuse than normal
- Other. Please specify

Are the people you live with understanding and/or supportive regarding issues or the negative impact related to your tinnitus (e.g. the inability to sleep or concentrate)?

- Not at all supportive
- Somewhat supportive
- Very supportive
- I live alone

Has understanding or support regarding your tinnitus from the people you live with changed since the coronavirus?

- Less supportive/ understanding
- No change
- More supportive/ understanding
- I live alone

Have you sought additional professional support for managing your tinnitus, after the onset of the coronavirus pandemic? Select all that apply

- No
- Contacted a helpline or chat service
- Been in contact with my usual clinic for advice
- Continuing with ongoing support from my usual clinic
- Received remote support from a professional (e.g. a skype or telephone consultation) free of charge
- Received remote support from a professional (e.g. a skype or telephone consultation) that I paid for
- Searched on the internet for advice
- Please provide information of who you contacted e.g. The British Tinnitus Association ________________________________________________

How helpful has this support been?

- Not helpful
- Somewhat helpful
- Very helpful
- Other. Please explain: ________________________________________________

Are you concerned that you will be unable to seek help for other health-related issues (not related to the coronavirus), because of the demands on hospitals caring for those with coronavirus? Select all that apply:

- I am not concerned
- Worried I cannot get help with my hearing aids or hearing-related issues
- Concerned that I cannot speak to a professional
- Concerned that I cannot contact a helpline
- Worried I cannot seek help for other health-related issues
- Worried I cannot seek help for other mental-health-related issues

What type of support would help you better manage your tinnitus or hearing related issues during these uncertain times?

________________________________________________________________

________________________________________________________________

________________________________________________________________

________________________________________________________________

________________________________________________________________

What advice/suggestions for healthcare professionals and/or researchers can you offer so that we might provide/develop more effective care for the future?

________________________________________________________________

________________________________________________________________

________________________________________________________________

________________________________________________________________

________________________________________________________________

Please provide any further information that may be helpful that has not been included in this survey

________________________________________________________________

________________________________________________________________

________________________________________________________________

________________________________________________________________

________________________________________________________________
